# Protective Effect of ALA in Crushed Optic Nerve Cat Retinal Ganglion Cells Using a New Marker RBPMS

**DOI:** 10.1371/journal.pone.0160309

**Published:** 2016-08-09

**Authors:** Yanling Wang, Wenyao Wang, Jessica Liu, Xin Huang, Ruixing Liu, Huika Xia, Nicholas C. Brecha, Mingliang Pu, Jie Gao

**Affiliations:** 1 Department of Anatomy, School of Basic Medical Sciences, Peking University, Beijing, China; 2 Key Laboratory on Machine Perception (Ministry of Education), Peking University, Beijing, China; 3 Key Laboratory for Visual Impairment and Restoration (Ministry of Education), Peking University, Beijing, China; 4 Department of Neurobiology and Molecular, Cellular and Integrative Physiology, UCLA, Los Angeles, California, United States of America; 5 Jules Stein Eye Institute, UCLA, Los Angeles, California, United States of America; 6 UCLA College of Life Sciences, University of California, Los Angeles, CA, United States of America; National Eye Centre, UNITED STATES

## Abstract

In this study we first sought to determine whether RNA-binding protein with multiple splicing (RBPMS) can serve as a specific marker for cat retina ganglion cells (RGCs) using retrograde labeling and immunohistochemistry staining. RBPM was then used as an RGC marker to study RGC survival after optic nerve crush (ONC) and alpha-lipoic acid (ALA) treatment in cats. ALA treatment yielded a peak density of RBPMS-alpha cells within the peak isodensity zone (>60/mm^2^) which did not differ from ONC retinas. The area within the zone was significantly enlarged (control: 2.3%, ONC: 0.06%, ONC+ALA: 0.1%). As for the 10-21/mm^2^ zone, ALA treatment resulted in a significant increase in area (control: 34.5%, ONC: 12.1%, ONC+ALA: 35.9%). ALA can alleviate crush-induced RGC injury.

## Introduction

The retina ganglion cell layer (GCL) consists mainly of retinal ganglion cells (RGCs) and displaced amacrine cells. Over the years many efforts have been made to develop methods to objectively differentiate between RGCs and displaced amacrine cells. Several antigenic RGC markers, including Brn3a [1–3], Thy-1[4], neurofilament [5], and retrograde labeling have been viewed as good markers for RGCs. It was reported that a member of the RNA recognition motif family of RNA-binding proteins known as RNA-binding protein with multiple splicing (RBPMS), and its paralogue RBPMS2 (hermes), are expressed in RGCs in rats [6–10]. Recent studies have revealed that RBPMS can label all RGCs in normal retinas of mouse, rat, guinea pig, rabbit, and monkey [11]. In addition, RBPMS can also serve as a RGC marker for quantitative analysis in animal models of RGC degeneration induced by IOP elevation, optic nerve crush, and excitotoxicity [11, 12–13]. The morphological and physiological properties of cat RGCs have been thoroughly investigated. However, whether RBPMS are a good marker for cat RGCs remains to be determined.

Although the neuromechanisms underlying glaucoma-induced RGC apoptosis remain controversial, increased levels of reactive oxygen species (ROS) are thought to play a crucial role in pathogenesis. A substantial body of evidence suggests that ROS are part of the signaling pathway in cell death after axonal injury. RGC axons within the globe are functionally specialized and are richly endowed with mitochondria. Mitochondria are important in the maintenance of cellular homeostasis as they are involved in numerous metabolic and physiologic functions. Mitochondria produce the energy required for nerve conduction in the unmyelinated part of ganglion cell axons. Thus, optic nerve injury-induced RGC apoptosis may at least partially be due to mitochondrial malfunction [14–15]. In this study, an optic nerve crush (ONC) model was used to examine RGC apoptosis. This ONC model has been widely used in studying the pathophysiology of glaucoma[16–19]. ALA is a disulfide compound found naturally in mitochondria that serves as the coenzyme involved in carbohydrate utilization necessary for the production of mitochondrial ATP. A substantial body of evidence shows that ALA is a superb antioxidant that enhances mitochondrial function [14, 20]. ALA provides protection to the retina as a whole, and to ganglion cells in particular in ischemia-reperfusion injuries [21] and optic nerve crush [14]. Recent studies have revealed that ALA exerts a neuroprotective effect against oxidative stress in retinal neurons [22–23]. Despite such positive results, the effectiveness of ALA as a neural protectant in the retina has not been investigated.

The purpose of the current study was to determine: (1) whether RBPMS can be used as a selectively marker for RGCs in the cat retina; and (2) whether ALA can alleviate ONC-induced RGC injury.

## Materials and Methods

### Animals

Fourteen young adult domestic cats of both sexes with body weights of 2.2 to 3.5 kg were used in this study. Two cats were used for testing RBPMS antibody and twelve cats were used for study of the effect of ALA. Animals were purchased from a local research animal provider(Xinglong Institute for Experimental Animals, Beijing; registration number:110108600078158. This is an ordinary animal housing facility and it managed in keeping with national standards as described in ⟪Laboratory Animal–Requirements of Environment and Housing Facilities⟫ (GB 14925–2001). The care of laboratory animals and animal experimental guidelines used in this study conformed to the ⟪Beijing Administration Rule of Laboratory Animal⟫. Each animal was housed in an individual stainless steel cage (dimensions 100cm ×100cm ×113cm) under a 12-hour light/12-hour dark cycle. A feeding station, water fountain, litter box, scratching post and pet toys were provided in each cage. Room temperature was set at 18–21°C. Commercial cat food and water were provided *ad libitum*. Procedures were approved by the Institutional Animal Care and Use Committee (IACUC) at Peking University, and all procedures adhered to the ARVO Statement for the use of animals in ophthalmic and vision research.

### Animal preparation and surgical procedures

#### Retrograde labeling

Two cats were anesthetized with intramuscular injections of ketamine (20 mg/kg) and xylazine (5 mg/kg) and placed in a stereotaxic instrument. Animals were kept warm with a heat lamp throughout the experiment. The superior colliculus (SC) was targeted as follows: a small hole was made using a dental drill (3 mm posterior to lambda; 2 mm to midline) and a 10ul Hamilton syringe was inserted vertically to a point 14 mm below the surface of the brain. A total of 1.5ul of RED Retrobeads (Lumafluor, Inc, Durham, NC) was slowly injected over 5 minutes and slowly withdrawn 10 minutes later. The wound was sutured and topical analgesic gel was applied three times a day for three days. Penicillin sodium (16000 U/kg) dissolved in sterile saline was then injected intramuscularly on a daily basis for three days to prevent infection. No animal in this study became ill or contracted an infection prior to the endpoint of the experiments.

#### Optic nerve crush (ONC)

Twelve cats underwent unilateral ONC. ONC surgery was performed following the procedure described by Weber and colleagues [24]. Briefly, cats were anesthetized as described above. The head of each animal was stabilized and the dorsal surface of the skull was shaved. Using sterile procedures, after the roof of the bony orbit was opened, the optic nerve sheath of the left eye was exposed in all treatment groups by blunt dissection of the overlying tissues without disturbing the nerve sheath or retinal artery. The left optic nerve was crushed 2 mm distal to the globe for 2 minutes with a 40 g vascular clamp (TKF-5-40, ARO Surgical Corp, Newport Beach, CA). The contralateral eye did not undergo any surgical procedure and served as a normal control. In all cases, the retinal blood supply remained grossly intact, as evaluated by direct ophthalmoscopic inspection. Observation of the fundus showed normal retinal vasculature. After removal of the clamp, penicillin sodium powder was applied topically, and the overlying tissue and skin were sutured and treated with betadine solution. The entire surgery required 30 minutes. Topical analgesic gel and penicillin sodium were applied as described above. Animal respiration rates and body temperature were monitored three times a day for three days after the surgery, and activity patterns were monitored 24 hours per day for seven consecutive days. One of the senior investigators (MPU) had a Peking University IACUC approved protocol in place (LA-2011-054) for the use of humane endpoints and early euthanasia for animals that became severely ill prior to the experimental endpoint. The criteria used to determine when to euthanize animals were based primarily on responses to pain. Responses to pain were judged by external physical appearance, clinical signs, unprovoked behavior, and behavioral responses to external stimuli. Two animals that experienced severe pain that could not be relieved by analgesic treatment for two consecutive days were euthanized.

#### Alpha-lipoic acid (ALA) application

Cats underwent ONC and were divided into two groups: five cats received no ALA treatment, and five cats received a single intravenous injection of ALA (15 mg/kg) (STADA, Germany) through the femoral vein immediately after ONC. Tissues from five cats with ONC alone, and from five cats with ONC and ALA treatment were analyzed.

### Tissue Processing

After an interval of 5 days to allow for retrograde labeling surgery and 7 days for ONC surgery, the animals were deeply anesthetized as described above. The retina preparation process was similar to that previously reported [24]. Animals were euthanatized with an intravenous injection of pentobarbital sodium (>50 mg/kg) and perfused transcardially with 0.75 L of physiologic saline (0.9%), followed by 1.0 L of solution containing 4% paraformaldehyde (PFA) (#15710, Electron Microscopy Sciences, Ft. Washington, PA, USA) in 0.1 M phosphate buffered saline (PBS)(pH 7.4, Sigma-Aldrich, USA). The brains were placed in the same fixative for subsequent processing. The eyes were enucleated, and the lens and vitreous were carefully removed in 0.1 M cold PBS. The eyecups were fixed immediately by immersion in 4% PFA as described above for 60 min at room temperature. The retinas were then dissected from the retinal pigment epithelium, and 3–4 radial cuts were made to flatten the retina. The retina was then mounted on a nitrocellulose membrane filter (GSWP02500, Millipore, USA) and transferred to a 6-well cell culture cluster (#3516, Corning Incorporated, Corning, USA) for immunohistochemistry staining.

### RNA binding protein with multiple splicing (RBPMS) antibody generation

A rabbit polyclonal antibody was generated against the N-terminus of the RNA Binding Protein Multiple Splice (RBPMS) polypeptide (RBPMS4-24), GGKAEKENTPSEANLQEEEVR by a commercial vendor (ProSci, Poway, CA). RBPMS is highly conserved among mammals and the polypeptide sequence used for immunization is identical in mouse, rat, monkey and humans (NCBI Protein Bank, http://www.ncbi.nlm.nih.gov/protein). Rabbit sera were collected following immunization and affinity purified using a RBPMS polypeptide affinity column. The affinity purified antibody was shown to immunostain ganglion cells in mouse and rat retina [11]. To evaluate the specificity of the RBPMS immunostaining, a preabsorption control was performed with the rabbit antibody. Briefly, RBPMS antibody was diluted in 0.1 M PB containing 0.5% Triton X-100 and mixed with RBPMS polypeptide at a final concentration of 1 μg/ml for two hours at RT. No RBPMS immunostaining was present in tissue sections incubated with the rabbit antibody preabsorbed with RBPMS and processed by standard immunohistochemical techniques.

### Antibodies and reagents

The primary antibodies used in this study were rabbit polyclonal antibody against RBPMS (1:1000) and goat polyclonal antibody against Choline Acetyltransferase (CHAT) (1:200, Cat# AB144P, Millipore, LOT#2070392, RRID:AB_2079751). The secondary antibodies were DyLight 549 anti-rabbit IgG, DyLight 488 anti-rabbit IgG (1:2000, Vector Labs, Inc., Burlingame, CA, USA), Alexa Fluor 488-conjugated AffiniPure Donkey anti-goat IgG (1:400, code: 705-545-147, Jckson Immuno Research, USA), and Dylight 549 conjugated Donkey anti-rabbit IgG (1:2000, code: 611-742-127, Rockland Immunochemicals Inc.,USA). 2%Triton X-100 (Sigma-Aldrich, USA) and 0.5% dimethylsulfoxide (Sigma-Aldrich, USA) in 0.1 M PBS (Sigma-Aldrich, USA) were used to facilitate antibody penetration. Blocking reagent (CAS-Block, Invitrogen Corp, US A) was used to suppress non-specific immunoreactions. The dilutions used to dissolve antibodies were 10% CAS-Block, 2% Triton X-100 and 0.5% dimethylsulfoxide in 0.1 M PBS.

### Immunohistochemistry

The retinas were washed six times for 10 minutes with 0.1 M PBS on a shaker at room temperature, blocked for 48 hours at 4°C, and incubated in primary antibody solution for 7 days at 4°C. After rinsing six times as described above to remove excess primary antibody, the retinas were incubated in secondary antibody overnight at 4°C. After washing an additional six times as described above, the free floating retinas were mounted on slides, immersed in anti-fade mounting medium (#17985–11, Electron Microscopy Sciences, Hatfield, PA) and covered with a cover slip (#12-545G, Electron Microscopy Science, USA).

### Imaging processing and quantitative morphological analysis

Entire whole-mount retinas were photographed with a 2.5× objective (3207×2415 μm²/microscope field), a 10×objective (872 ×65403BCm²/microscope field) and a 20×objective (436×327 μm²/microscope field) under fluorescent microscopy (BX51, Olympus, Japan). In each sampling field, RGCs were labeled for RBPMS immunoreactivity.

Photographic images were montaged with Adobe Photoshop CS5 graphic software (Adobe Systems, Inc., San Jose, CA, USA). The average soma densities of RBPMS expressing RGCs at different eccentricities were calculated with Photoshop. RBPMS labeled RGC counts were determined from retinal whole-mounts and were converted to cells per square millimeter. For the cell density versus spatial quantification analysis of normal retina whole-mounts, each retina was divided into superior, inferior, nasal and temporal quadrants. Twenty predefined areas (436×327 μm^2^/microscope field) with a separation of 1000 μm were analyzed from the central to the peripheral retina. Location of the area centralis (AC) was selected as the zero retinal eccentricity point. The twenty sampling areas were distributed along the vertical axis. To compare RGC survival under three different conditions (normal, ONC with or without ALA treatment), we divided the retina whole mounts as described above and selected only ten areas with six areas next to the AC in the superior quadrants and four areas next to the AC in the inferior quadrants. Ratios of total numbers of surviving RGCs in these ten areas of the ONC eye to those in the normal control eyes were obtained to estimate the survival rate of injured RGCs. Cell counting was carried out in a double-blinded manner. The final images were processed and saved in TIFF format at 300 dpi using Photoshop software. To analyze the alpha-RGC distribution pattern under these three different conditions, the AC was selected as the geometric center of the retina, and from this point the entire retina was divided into 8×8 grids, each grid covering a 2 mm×2 mm retinal area. The sampling area at the center of each grid was 872×654 μm^2^, and the density was determined by dividing the total number of RBPMS expressing RGCs identified within this area by 0.57. An isodensity map of retinal RBPMS expressing alpha cells was generated using the Contourf function of Matplotlib software [25]. The contour plot was generated by assigning a color code to each of the 64 grids according to its RBPMS expressing alpha cell isodensity value (density/mm^2^) within a six color scale ranging from 0 (camel) to 60, representing a higher isodensity region (navy blue).

### Statistics

All results are reported as mean ± standard error (SE). Statistical significance was calculated using the Students’ T-test for comparisons between two groups, and one-way ANOVA for multiple comparisons followed by the SNK test as a post hoc test with SPSS 13.0 for Windows Software (SPSS Inc., Armonk, NY, USA). Values of P<0.05 were regarded as significant in all comparisons.

## Results

### Retrograde labeling and double fluorescence immunohistochemistry

**Fig 1:**
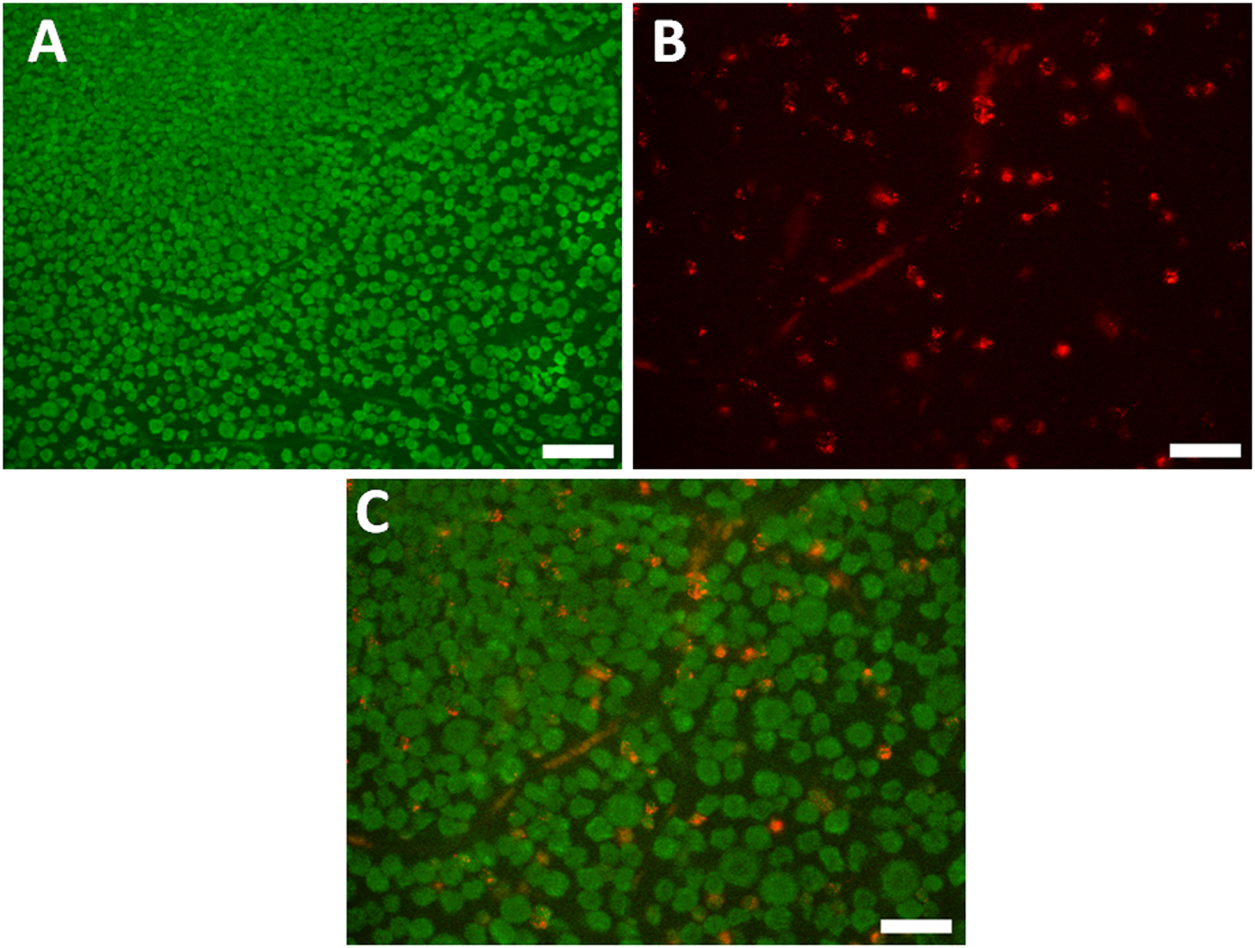
Double labeling with RBPMS, and retrograde RGC labeling with Microbeads. A: RBPMS positive cells in a normal retina. B: Microbead labeled RGCs. C: Co-localization of RBPMS (green) and Microbeads (red). Scale bar: 50 μm.

**Fig 2:**
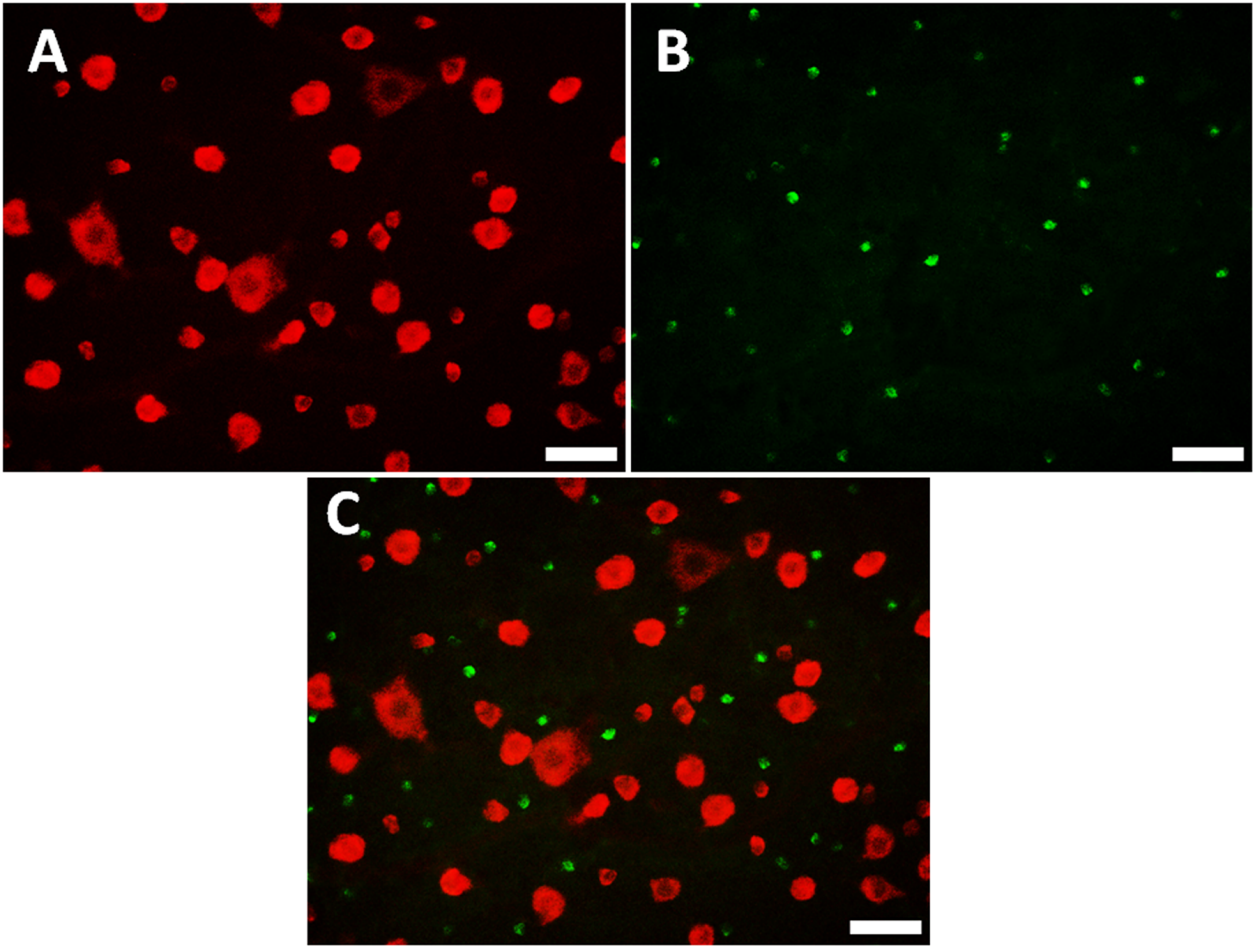
Double labeling with RBPMS and CHAT. A: RBPMS positive cells in a normal retina. B: CHAT positive cells. C: Co-localization of RBPMS (red) and CHAT (green). Scale bar: 50 μm.

At least eight microscopic fields from each of two retinas were included for quantitative analysis. 100% of the cells labeled by microbeads (Fig 1) were immunoreactive for RBPMS, while only 15.8± 0.9% (range 14.8% to 17%) of RBPMS positive cells were labeled by microbeads (Table 1). No RBPMS positive cell expressed CHAT (Fig 2) in the GCL.

**Table 1:** Quantification of double labeling with microbeads and RBPMS in whole mount retinas.

	Retina1	Retina2
% of RGCs (beads) are RBPMS-positive	100	100
% of RBPMS-positive cells are RGCs (beads)	15.8	15.9

**(**n = 2. eight images per retina and sixteen images in total)

### The distribution patterns of RBPMS expressing RGCs in cat retinas

**Fig 3:**
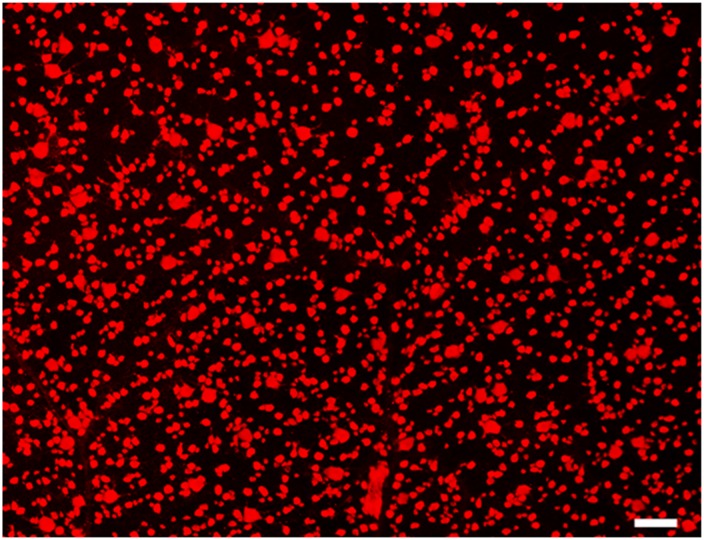
Micrograph of RBPMS expressing RGCs from a normal cat retina. Micrograph location: 2.5 mm temporal and 1.6 mm superior to the AC. Scale bar: 100 μm.

**Fig 4:**
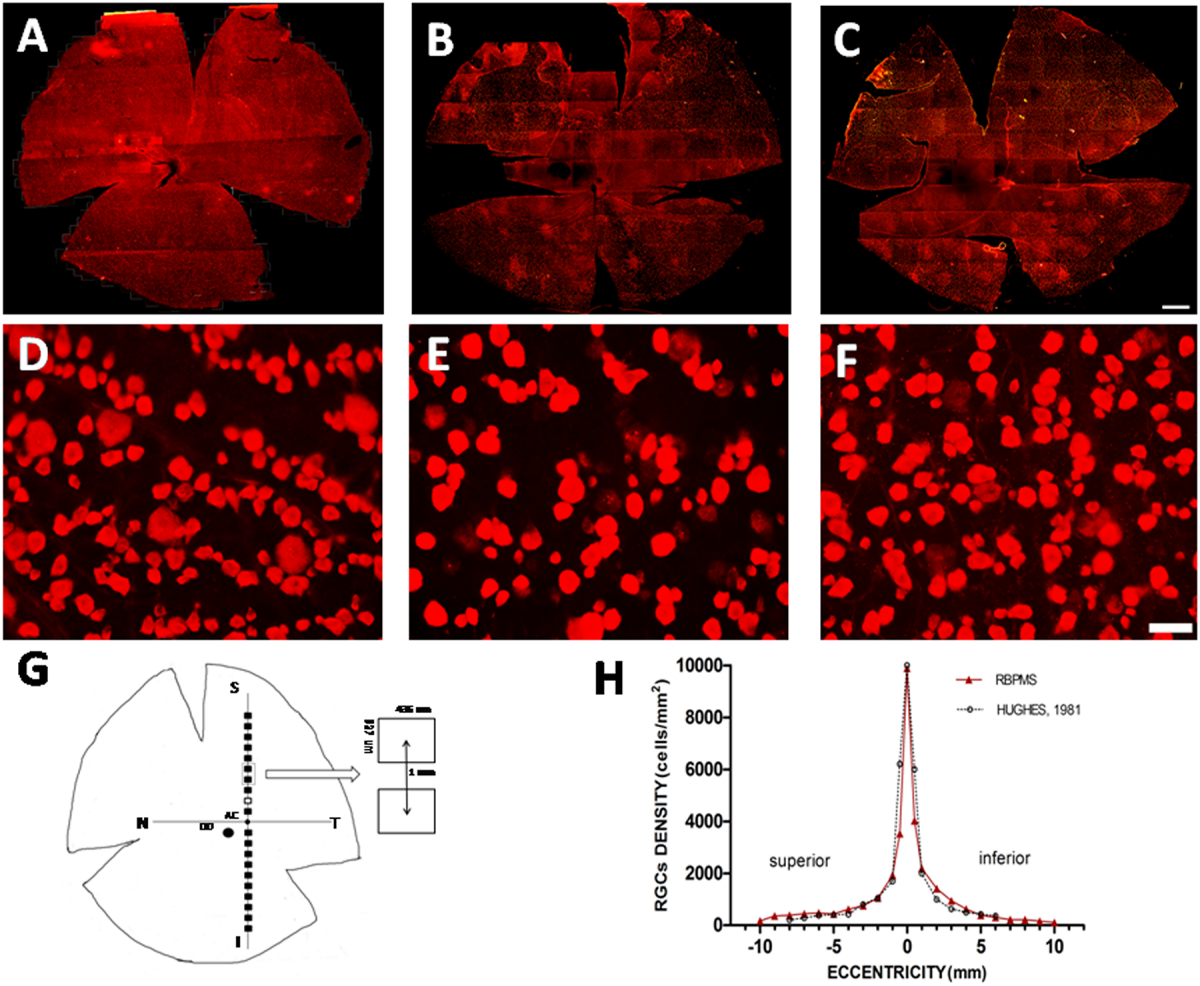
Number and density distribution of RBPMS-RGCs under different conditions. A-C reveal retinal distribution patterns of RBPMS expressing RGCs under different experimental conditions. A: RBPMS expressing RGCs in a normal retina, B: ONC retina, C: ONC+ALA retina. Scale bar: 2 mm. D-F show magnified micrographs of RBPMS immunoreactive RGCs (Sampling location: 2 mm superior to the AC from A-C) under different experimental conditions: D: normal retina, E: ONC, F: ONC + ALA. Scale bar: 50 um. G shows a diagram of sampling sites along the vertical meridian on each retina. The open rectangle depicts retinal location where micrographs were taken. The filled rectangles show sampling locations at different eccentricities. The large filled circle represents the optic disk (OD) and the small filled circle at the intersection of horizontal and vertical meridians shows the AC. Panel H shows a comparison of RBPMS stained and HRP stained density profiles. The filled red triangles and solid line depict RBPMS. Open black circles and the dashed profile which is taken from Hughes (1981, his Fig 13), is shown for comparison. The open arrow indicates sample area dimension and the distance between centers of two sample sites. Each sampling area was measured as 436×327μm^2^. The distance between centers of neighboring sampling areas is 1 mm.

Retinal ganglion cells of various soma sizes and RBPMS-RGCs were distributed across the entire retina. Fig 3 shows a micrograph of typical immunocytochemically stained RBPMS-RGCs in a cat retina. We quantitatively analyzed the numbers and density of RBPMS-RGCs (Fig 4G and 4H). The density distribution reached a peak at the AC and gradually tapered down at the periphery of the retina (Fig 4H). This RBPMS-RGC distribution profile matches that of HRP labeled RGCs [26]. The numbers and distribution of RBPMS-RGCs were also quantitatively analyzed in whole mount retinas. One of the characteristics of RGC distribution in cats is that the density of RGCs change drastically within 1 mm of the center of the AC. Thus, we counted and verified this pattern of density distribution around the AC. The density of RBPMS-RGCs within 1 mm^2^ of the center of the AC was 5610/mm^2^, while density increased to 8174/mm^2^ when the sampling area was reduced to 0.0314 mm^2^. If the sampling area was reduced to 0.016 mm^2^, the density increased to 9043 mm^2^ (n = 4). Thus, the peak density of RBPMS-RGCs varied with sampling area with respect to the distance from the center of the AC. We then counted the numbers of RBPMS-RGCs at ten superior and ten inferior locations along the vertical meridian. The RMBMS density distribution profile matched that of the HRP stained RGCs, as shown in Fig 3H. We next compared the numbers and density of RBPMS-RGCs under different experimental conditions. The top panels of Fig 4 illustrate whole mount retinas under different experimental conditions (Fig 4A: Normal, Fig 4B: ONC, and Fig 4C: ONC with ALA treatment (ONC+ALA)). The distribution pattern of RBPMS-RGCs in a small area (encircled dashed square) of a normal whole mount retina is shown in Fig 3D, while Fig 4E and 4F illustrate RBPMS-RGCs after ONC and ONC+ALA, respectively. To quantitatively assess the impact of ONC on RGCs under these conditions, the number of RBPMS expressing RGCs was counted in ten sampling locations along the vertical meridian, six from the superior and four from the inferior retina as shown in Fig 4G. The total number of RBPMS-RGCs was counted and is summarized in Table 2. In comparison with the control (1466 ± 20/mm^2^), ONC resulted in a sharp reduction in the number of RPBMS-RGCs (1012 ± 38/mm^2^) while ALA treatment reduced the impact of ONC on RBPMS-RGCs (1178 ± 27/mm^2^).

**Table 2:** Average density of RGCs after ONC with or without lipoic acid treatment.

	NORMAL	ONC	ONC+ALA
Alpha-RGCs (cells)	68±4	29±4	41±4
total-RGCs (cells)	1466±20	1012±38	1178±27

(n = 5 for each group.)

### ALA improves the distribution patterns of alpha ganglion cells in the crushed optic nerve of cats

**Fig 5:**
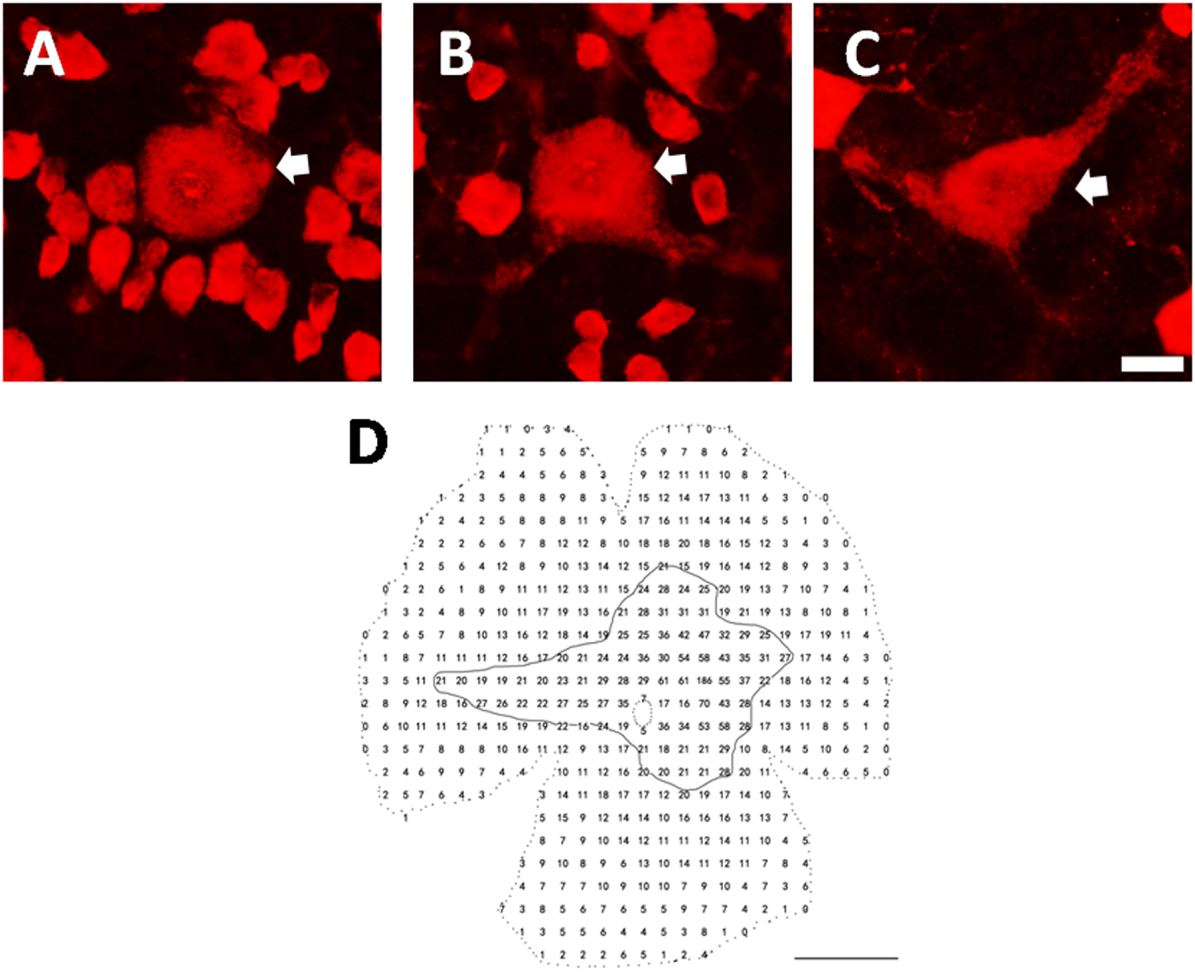
RBPMS expressing alpha cells and their density distribution pattern. A-C illustrate micrographs of RBPMS-alpha RGCs photographed at 1, 5, and 10mm from the AC. Scale bar: 20μm. Arrows depict alpha cells. D shows density distribution pattern of RBPMS-alpha RGCs from a normal cat retina. Each number represents the total number of alpha cells encountered within the sampling field. The size of sampling area was 1×1 mm^2^. The distance between centers of two neighboring sampling fields was 1 mm. The peak density at the AC was 186/mm^2^. The dashed circle in the center depicts the optic disk. The edge of the visual streak is defined as local alpha cell density greater than 20/mm^2^. The area encircled by the solid line represents the visual streak. Scale bar: 5 mm.

**Fig 6:**
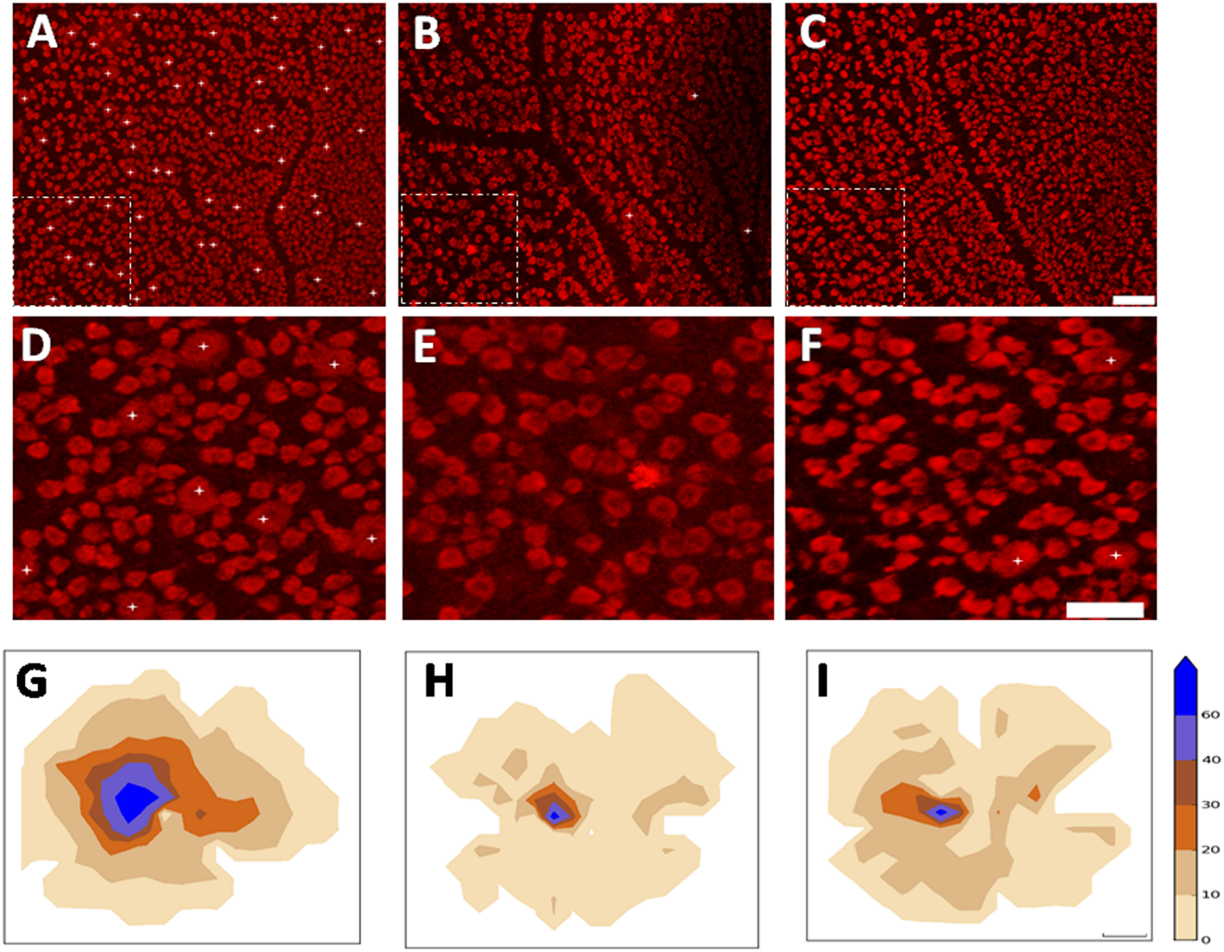
Number and density distribution of RBPMS-alpha RGCs under different experimental conditions. A-C show retinal micrographs taken 1 mm temporal to the AC in the superior retina. A: Normal, B: ONC, C: ONC+ALA. Scale bar: 100 μm. D-F show magnified areas in the panels above (dashed line enclosed areas). Asterisks denote alpha cells. Scale bar: 100 um. Bottom panels provide alpha cell isodensity distribution maps under different experimental conditions. G: Normal (n = 3), H: ONC (n = 3), and I: ONC+ALA (n = 4). Color scale ranges from 0 (camel) to 60 or higher RGCs/ mm^2^ (navy blue): 0–10 –camel; 11–20 –desert dune; 21–30 –light brown; 31–40 –brown; 41–60 –light blue, 61 and up—navy blue. Scale bar: 2 mm.

**Fig 7:**
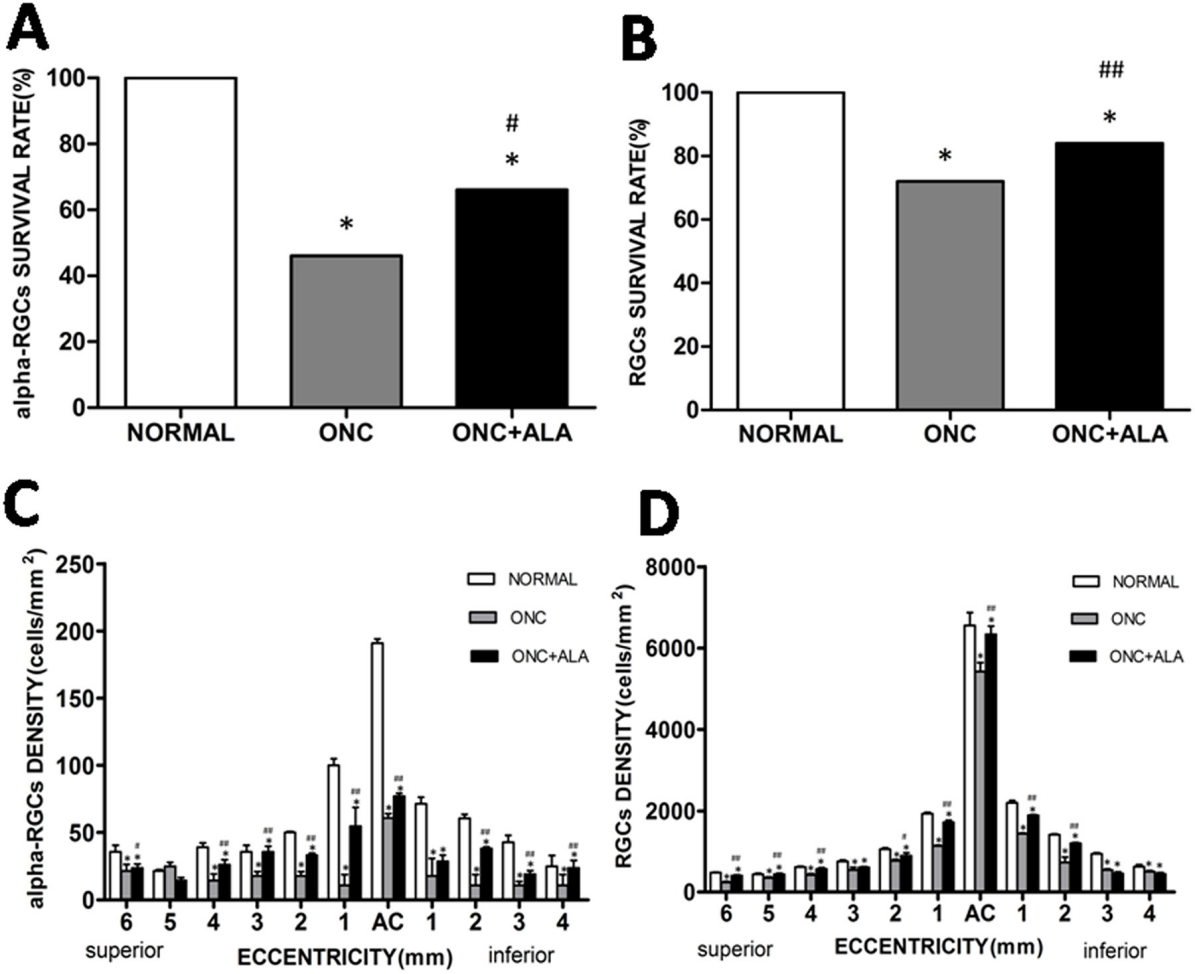
Histograms of survival rate and density distribution of RBPMS-alpha cells and RBPMS-RGCs under different experimental conditions. A: Survival rate of alpha-RGCs, B: Survival rate of RGCs, C: Density distribution of alpha cells at different eccentricities, D: Density distribution of RGCs at different eccentricity. Mean ± SE (n = 5, *compared to normal P<0.001, # compared with ONC, #P<0.05,## P<0.01).

One of the morphological characteristics of alpha cells is their gigantic soma. RBPMS expressing alpha cells (RBPMS-alpha cells) were thus defined by giant sized perikaryon (>20 μm at AC and >35 μm, in the visual streak at the peripheral retina), and alpha cells with soma size that is always larger than any of the RGCs at different eccentricities. As RBPMS antibody staining is present in the perinuclear zone of medium sized soma, and is found predominantly in the cytoplasm of large sized soma, the gigantic cell body of alpha cells is thus readily identifiable. Fig 5A–5C shows RBPMS-alpha cells. These cells were photographed at different eccentricities (Fig 5A: 1 mm; Fig 5B: 5 mm; and Fig 5C: 10 mm). Fig 5D shows the distribution pattern of alpha cells in a normal retina. Each number represents the total number of alpha cells found within a sampling field of 1 mm^2^. The distance between the centers of two neighboring sampling fields was 1 mm. The density distribution of alpha cells was measured across the entire retina, and the peak density at the AC was 186/mm^2^. Along the horizontal meridian, the density was reduced to 61/mm^2^ two millimeters from the AC on the nasal retina and to 37/mm^2^ on the temporal retina. Along the vertical meridian, the density was reduced to 47/mm^2^ two millimeters from the AC on the superior retina and 53/mm^2^ on the inferior retina. Cell density gradually tapered down along the vertical and horizontal meridians. We defined the edge of the visual streak as the retinal region where the alpha cell density was greater than 20/mm^2^. The area encircled by the solid line represents the visual streak (Fig 5D). Because cat retinal alpha/Y cells are more susceptible to optic nerve injury than other RGCs [27–28], we first observed the impact of ONC on RBPMS-alpha cells. The top panels (Fig 6A: normal, Fig 6B: ONC, Fig 6C: ONC+ALA) show images taken from the superior retina, 1 mm from the AC. We then compared RBPMS-alpha cells at a given retinal location (area enclosed by a dashed line). Panels D through F show enlarged micrographs of the sampled areas (256× 256 μm^2^) under different experimental conditions (Fig 6D: Normal, Fig 6E: ONC, and Fig 6F: ONC + ALA). Asterisks denote RBPMS-alpha cells. ONC completely eliminated RBPMS-alpha cells (Fig 6E, 13/mm^2^) compared to nomal retina (6D, 70/mm^2^), while ALA treatment substantially prevented ONC-induced injury to these cells (Fig 6F, 21/mm^2^). We next investigated the global impact of ONC on RBPMS-alpha cells. As shown in the bottom panels (Fig 6G–6I), the isodensity profiles of RBPMS-alpha cells reveal different distribution patterns after ONC. It is evident that ONC resulted in a substantial shrinkage of the high density alpha cell distribution area (Fig 6H). Although ALA treatment did not change the alpha cell peak density at the AC (control: 186/mm^2^, ONC: 67/mm^2^, ONC+ALA: 69/mm^2^), the sizes of both the high isodensity zones (>60/mm^2^) and low density regions (0–10 mm^2^ and 10-21/mm^2^) were substantially recovered (Fig 6 and Table 3, data in S1 Table). Finally, the survival rates of RBPMS-alpha and RBPMS-RGCs were analyzed (Fig 7A and 7B). Except for one superior retinal location (eccentricity = 5 mm), the density distributions at every measured site showed a sharp decline and ALA improved the density distribution of alpha cells (Fig 7C). As for the RBPMS-RGCs, results from three out of eleven measured sites show that ONC reduced the density, and ALA improved the local density distribution of RBPMS-RGCs (Fig 7D). Together, these results suggest that alpha cells are more sensitive to ALA treatment than other classes of RGCs after ONC.

**Table 3:** Alpha cell isodensity distribution under different experimental conditions.

Density(cells/mm^2^)	NORMAL (%)	ONC (%)	ONC+ALA (%)
0–10	33.6±4.28	85.6±1.72	59.7±8.47*
11–20	34.5±1.71	12.1±1.80	35.9±7.11**
21–30	17.4±0.81	1.13±0.12	2.84±1.21
31–40	7.10±1.38	0.60±0.18	0.81±0.18
41–60	5.03±0.46	0.50±0.02	0.66±0.14
>60	2.35±0.39	0.06±0.01	0.10±0.02*

*(normal*, *n = 3; ONC*, *n = 3; ONC+ALA*, *n = 4*. Mean ± SE,

*compared to ONC, *, P<0.05

**, P<0.01)

## Discussion

In this study fourteen cats were used, including two cats for demonstrating that RBPMS selectively labels all cat RGCs. Twelve cats were used to study the impact of ONC-induced neurodegenerative changes on RGCs and the neuroprotective effects of ALA, and two of the cats in this group were euthanized before the endpoint of the experiment. Retinas from remaining ten cats were divided into two groups (ONC alone and ONC+ALA) and were used for data analysis. One or two retinas in each group were partly damaged during the tissue processing, and were inadequate for some data analysis. The sample size of each group therefore varied.

Numerous efforts have been made to develop methods to selectively and reliably differentiate between RGCs and displaced amacrine cells. However, selective RGC markers remain to be identified. Evidence has demonstrated that RBPMS can selectively label retinal ganglion cells across several mammal species [7, 11–12, 29], but there has been no report of RBPMS labeling RGCs in cats. In this study, we used microbead retrograde labeling and RBPMS staining to determine whether cat RGCs can be labeled by RBPMS. 100% of retrograde labeled RGCs were RBPMS positive, illustrating that RBPMS label all RGCs in the cat retina. In this study, we observed that retrograde labeled RGCs are located mainly in a small part of the retina whole mount, which may explain why only 15.8% of RBPMS positive cells were labeled by microbeads from the SC. Tracer may not spread through the entire SC, and injecting tracer into SC may thus be insufficient for labeling all RGCs. We also tested CHAT which marks displaced amacrine cells and RBPMS double labeled retina whole mounts. No RBPMS positive cells were labeled with CHAT, showing that RBPMS is a reliable marker which can distinguish RGCs from displaced amacrine cells. In addition, we compared the total number and peak density of RBPMS immunoreactive RGCs with RGCs identified by classical cresyl violet or methylene blue staining [30–32] and HRP retrograde labeled RGCs [26]. Over the years, several attempts have been made to accurately determine the peak density of RGCs at the center of the AC. However, the density varies from less than 6000/mm^2^ [30] to over 10000/mm^2^ [26, 32]. This variation may be due to differences among animals [31]. In addition, the actual size of the counting area may be crucial, as peak density drops sharply within the AC. For example, the density is less than 6000/mm^2^ if the counting area is 1 mm^2^ [30], whereas it is over 10000/mm^2^ if the area is reduced to 0.0089 mm^2^ [26]. Consequently, we found RBPMS-RGCs within 1 mm^2^ of the center of the AC have a density of 5610/mm^2^, while the density increased to 8174/mm^2^ when the area was 0.03144mm^2^, and if the sampling area was reduced to 0.016 mm^2^, the density further increased to 9043 mm^2^ (n = 4). These numerical findings closely resemble previous observations (9040/mm^2^, [26], 8788/mm^2^, [32]). We thus confirmed that the peak density of RBPMS labeled RGCs matches previously reported results that were obtained using conventional staining methods. We next quantitatively analyzed alpha cells. As shown in Fig 4, the total number of RBPMS-alpha cells was 5682. This number closely resembles the number derived by HRP staining (5600, [33] and is in agreement with results of both Wässle and colleagues [32] and Stone [31]. As for the peak density of RBPMS-alpha cells in the AC, our estimation of 186/mm^2^ (n = 3) is comparable to previous reports (195/mm^2^, [32]; 186/mm^2^, [31]. We thus demonstrate that RBPMS can be a selective RGC marker for cat cells as well as for other mammalian species [11]. RBPMS expression in RGCs was first demonstrated by analyzing its transcription with RT-PCR and in situ hybridization [7]. These authors observed very few RGCs expressing RBPMS in axotomized retinas. Reduced RBMPS expression levels in RGCs can be observed in different optic nerve injury animal models [11–13]. Taken together, these results suggest that RBPMS can be used to identify cat RGCs.

There is a substantial body of evidence that large soma RGCs are vulnerable to injury in humans and monkeys [34–37]. In addition, numerous physiologic and morphologic results reveal that Alpha/Y cells are more sensitive to optic nerve injury than other RGCs in cats [27–28, 38–40] and canines [34]. There is evidence that Beta/X cells are more sensitive to injury than Alpha/Y cells [41–42]. Nevertheless, there is a significant decrease in the soma, dendritic field size, and dendritic complexity of alpha cells, while beta cells display only a significant decrease in soma size [39]. It was reported that up to 55% of RGCs located on the periphery of the retina and 47% around the AC survived one week after ONC, and BDNF treatment drastically improved survival rates to 91% in both of these locations [40]. Contrary to this observation, we found substantial shrinkage of the topographic isodensity zones of RBPMS-alpha cells, especially in high density areas, and there was a 70% drop in the 10-21/mm^2^ isodensity zone seven days after ONC. However, as shown in Fig 6, the peak density area around the AC showed significant recovery (see Table 3), and two low density zones also recovered. For example, the 10-21/mm^2^ zone had a full recovery. The full recovery of this low density zone could due to several factors. One possibility is based on the speculation that RGCs in high density zones consume more oxygen and are thus more sensitive to ONC, while RGCs in low density zones consume less oxygen and better tolerate ONC-induced injury. ALA is therefore an effective antioxidant in the short term, as it alleviated the stress of RGCs in low density zones. A second point of speculation is that the recovered 10-21/mm^2^ zone occupied some of the areas that were high density zones prior to the injury. Indeed, the observed 0-10/mm^2^ isodensity zone showed similar recovery trends. The greater retinal space occupying area of this zone may be due to massive RGC injury and recovery after ONC. Thus, to quantitatively and objectively evaluate the impact of ALA treatment, it would be important to verify visual functions of RGC undergoing recovery within different density zones. In this study, axotomy-induced cell apoptosis resulted in decreased numbers of RBPMS positive neurons after ONC. However, this may also due to axotomy-induced RBPMS expression level down regulation that is difficult to recognize. In fact we observed that a small portion of RGCs in the ONC model were weakly labeled with RBPMS. Research has shown that optic nerve crush can produce a transient shrinkage of RGCs in rats [43]. A possible explanation for the decrease in numbers of alpha cells is that there is cell shrinkage after axonal lesions.

Although the neuromechanisms underlying glaucoma-induced RGC apoptosis remain controversial, increased levels of ROS are thought to play a crucial role in its pathogenesis. A substantial body of evidence suggests that ROS are part of the signaling pathway in cell death after axonal injury. RGC axons within the globe are functionally specialized and are richly endowed with mitochondria. These mitochondria generate the high energy required for fast optic nerve conduction in the unmyelinated part of RGC axons. Thus, optic nerve injury-induced RGC apoptosis may be at least partly due to mitochondrial malfunction [14]. The present study shows that ALA treatment improved RGC survival rate after ONC, which is consistent with previous reports showing that BDNF induces protection [39]. However, after ischemia-reperfusion, the retinal BDNF mRNA level was not upregulated by ALA [21]. Therefore, these two substances may act through different neuromechanisms. Nevertheless, recent studies have revealed that ALA exerts a neuroprotective effect against oxidative stress in retinal neurons [22–23]. Despite such positive results, the effectiveness of ALA as a neural protectant in the retina remains to be determined.

## Supporting Information

S1 Table Raw data of alpha cell isodensity area under different experimental conditions. (DOCX)
