# Supplementary material for: Protective Effect of ALA in Crushed Optic Nerve Cat Retinal Ganglion Cells Using a New Marker RBPMS
Source: PLoS One. 2016 Aug 9;11(8):e0160309. doi: 10.1371/journal.pone.0160309 (PMC4978478; doi:10.1371/journal.pone.0160309)
Supplement: S1 Table — (DOCX) [file pone.0160309.s001.docx]

S1 Table. Raw data of alpha cell isodensity area under different experimental conditions.

| Density(cells/mm^2^) |  | NORMAL | |  | |  |  | ONC+ALA | | |  | ONC | | | |
| --- | --- | --- | --- | --- | --- | --- | --- | --- | --- | --- | --- | --- | --- | --- | --- |
|  |  | 1 | 2 | | 3 |  |  | 1 | 2 | 3 |  | 1 | 2 | 3 | 4 |
| 0-10 |  | 25.6 | 40.2 | | 35.1 |  |  | 71.69 | 43.33 | 64.05 |  | 85 | 81 | 87.67 | 88.71 |
| 10-20 |  | 37.8 | 32.0 | | 33.8 |  |  | 26.67 | 49.87 | 31.15 |  | 12.8 | 16.9 | 9.12 | 9.55 |
| 20-30 |  | 19.0 | 16.3 | | 16.9 |  |  | 0.58 | 4.74 | 3.2 |  | 1.2 | 1.01 | 1.44 | 0.87 |
| 30-40 |  | 9.80 | 5.21 | | 6.29 |  |  | 0.45 | 0.98 | 1.01 |  | 0.44 | 0.44 | 1.13 | 0.37 |
| 40-60 |  | 4.69 | 4.46 | | 5.95 |  |  | 0.53 | 0.95 | 0.51 |  | 0.48 | 0.52 | 0.55 | 0.44 |
| >60 |  | 3.13 | 1.90 | | 2.03 |  |  | 0.08 | 0.13 | 0.08 |  | 0.03 | 0.08 | 0.08 | 0.06 |
